# Gabexate in the prophylaxis of post-ERCP pancreatitis: a meta-analysis of randomized controlled trials

**DOI:** 10.1186/1471-230X-7-6

**Published:** 2007-02-12

**Authors:** Minghua Zheng, Yongping Chen, Xinjun Yang, Ji Li, Youcai Zhang, Qiqiang Zeng

**Affiliations:** 1 Department of Infection and Liver Disease, The First Affiliated Hospital of Wenzhou Medical College, Wenzhou, China; 2 School of Environmental Science and Public Health, Wenzhou Medical College, Wenzhou, China; 3 Department of General Surgery, The First Affiliated Hospital of Wenzhou Medical College, Wenzhou, China

## Abstract

**Background:**

Acute pancreatitis is a common complication of endoscopic retrograde cholangiopancreatography and the benefit of its pharmacological treatment is unclear. Although prophylactic use of gabexate for the reduction of pancreatic injury after ERCP has been evaluated, the discrepancy about gabexate's beneficial effect on pancreatic injury still exists. This study aimed to evaluate the effectiveness and safety of gabexate in the prophylaxis of post-endoscopic retrograde cholangiopancreatography pancreatitis (PEP).

**Methods:**

We employed the method recommended by the Cochrane Collaboration to perform a meta-analysis of randomized controlled trials (RCTs) of gabexate in the prevention of post-ERCP pancreatitis (PEP) including three RCTs conducted in Italy and one in China.

**Results:**

All of the four RCTs were of high quality. When the RCTs were analyzed, odds ratios (OR) for gabexate mesilate were 0.67 [95% CI (0.31~1.47), p = 0.32] for PEP, 3.78 [95% CI (0.62~22.98), p = 0.15] for severe PEP, 0.68 [95% CI (0.19~2.43), p = 0.56] for the case-fatality of PEP, 0.88 [95% CI (0.72~1.07), p = 0.20] for post-ERCP hyperamylasemia, 0.69 [95% CI (0.39~1.21), p = 0.19] for post-ERCP abdominal pain, thus indicating no beneficial effects of gabexate on acute pancreatitis, the death rate of PEP, hyperamylasemia and abdominal pain. No evidence of publication bias was found.

**Conclusion:**

Gabexate mesilate can not prevent the pancreatic injury after ERCP. It is not recommended for the use of gabexate mesilate in the prophylaxis of PEP.

## Background

ERCP is one of the important procedures for the diagnosis and treatment of several biliary and pancreatic conditions. However, ERCP can also cause acute pancreatitis and result in significant morbidity and mortality [1,2]. Depending on the definition, it has been reported that the incidence of post-ERCP pancreatitis (PEP) was 1% to 40% of cases, whereas post-operative hyperamylasemia can be up to 70% of cases [3]. Although most cases of PEP were mild, there were still 10% of cases developing to severe pancreatitis, which could result in prolonged stay in the hospitals and increase the risk to patients' life.

Since the activation of proteases is one of the forms of the recognized PEP pathogenesis, agents that inhibit proteolytic activity were examined in several studies. Although a recent report indicated prophylactic treatment with gabexate could reduce pancreatic injury after ERCP [4], other studies reported marginal beneficial effect of gabexate on PEP [5,6]. The contradictory results of gabexate mesilate in the prophylaxis of PEP can only be resolved from large prospective randomized clinical trials (RCTs). However, a meta-analysis of all available RCTs will provide useful information for the use of gabexate mesilate in the prophylaxis of PEP. Since gabexate is used prophylactically to prevent pancreatic injury after ERCP in China, we included a RCT [7] conducted in Chinese population in current meta-analysis.

## Methods

### Selection criteria

We searched different databases, which included the Cochrane Controlled Trials Register on The Cochrane Library Issue 2, 2006, MEDLINE (January, 1966 – June, 2006), EMBASE.com (January, 1966 – June, 2006) and the China Biological Medicine Datadase (CBMdisc) (January, 1978 – June, 2006) by the terms of pancreatitis, ERCP, prevent*, gabexate, PEP. The reference lists of pertinent reviews and retrieved articles had been also checked for additional studies identification.

In the meta-analysis, the following inclusive selection criteria were set and reviewed by two independent investigators: (1) each trial should be a prospective randomized controlled clinical trial, (2) the age of patient population should be over 18 years, (3) the patients were scheduled to undergo ERCP and/or endoscopic sphincterotomy, (4) randomized comparisons of gabexate versus placebo or blank control should be included regardless of the initial time of treatment, treatment duration, dose and administration route of the drug, (5) co-interventions (including treatment of complications) were allowed if administered equally to all intervention groups. The following exclusive selection criteria were set: (1) quasi-randomized trials and non-randomized studies, (2) active acute pancreatitis, chronic pancreatitis, pancreatic cancer, or cancer of the papilla of Vater, (3) difference of co-interventions between intervention arms, (4) the raw data was not completed, (5) repetitive reports (if more than one version of the same study was retrieved, only the most recent was used).

**Figure 1:**
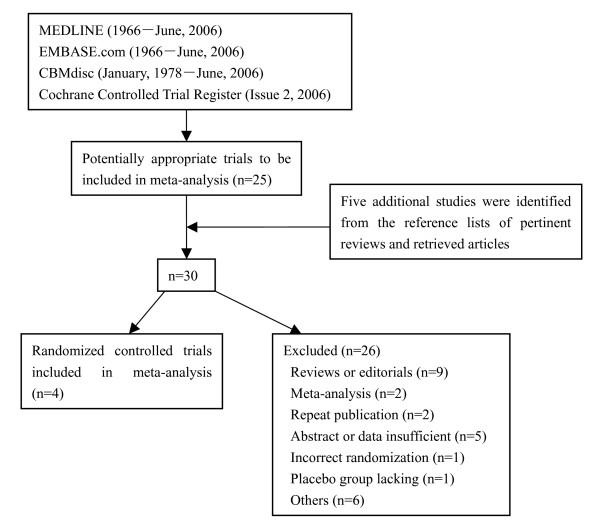
Identification of eligible randomized controlled trials from different medicine databases.

A total of 30 clinical trials and reports has been identified and only four trials [4,7-9] were qualified by our selection criteria (Figure 1). The studies were independently evaluated by two of us with five outcomes, which included three primary outcomes (PEP, severe PEP and the case-fatality ratio of PEP) and two secondary outcomes (post-ERCP hyperamylasemia and abdominal pain). Discrepancies in the evaluation of some of the studies were resolved through discussion between the reviewers. The main features of the trials included in the meta-analysis are shown in Table 1.

### Assessment of study quality

Quality of included reports was scored using the Jadad composite scale [10], which assesses the descriptions of randomization, blinding, and dropouts (withdrawals) in the report [11]. The quality scale ranges from 0 to 5 points with a low-quality report of score 2 or less and a high-quality report of score at least 3 [12]. The quality score of the four RCTs were shown in Table 2.

### Statistical analysis

The meta-analysis was carried out by a biostatistician (X.Y.) according to the Cochrane Reviewers' Handbook recommended by The Cochrane Collaboration. Pooled odds ratio (OR) was calculated using the general inverse variance (IV) fixed-effect model. The heterogeneity between studies was examined by DL Q statistic [13]. If results were heterogeneous (p < 0.05), a random-effects model was employed using the DerSimonian and Laird (DL) methods. Pooled OR was presented as standard plots with 95 percent confidence intervals (CI). Begg and Mazumdar's proposed adjusted rank correlation test [14] and Egger's linear regression approach [15] were used to measure publication bias, which was shown as a funnel plot. Sensitivity-analysis was also performed to assess the reliability of meta-analysis. The statistical package RevMan version 4.2 (provided by The Cochrane Collaboration, Oxford, England) was used for the statistical analysis.

## Results

### Primary outcome

**Figure 2:**
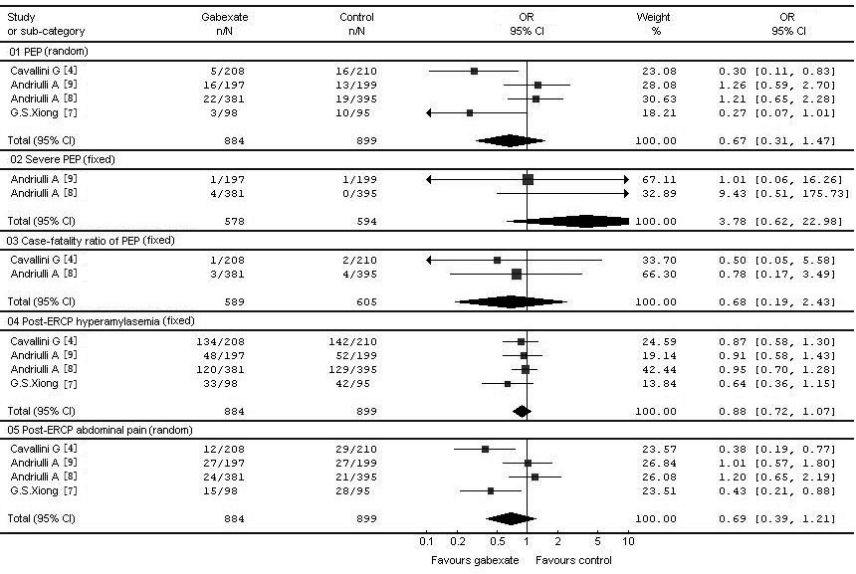
Effect of gabexate on the frequency of ERCP-related acute pancreatitis, severe pancreatitis, case-fatality ratio, post-ERCP hyperamylasemia and post-ERCP abdominal pain.

In this report, we considered PEP as the primary outcome which was divided into general PEP and severe PEP. The report of general PEP was noticed in all four RCTs [4,7-9]. These trials included 1783 patients with 104 patients suffering from PEP. Among PEP-suffering patients, 46 patients were treated with gabexate whereas 58 patients were treated with placebo. There was a significant heterogeneity among these studies (Q = 9.26, 3 degrees of freedom, p = 0.03). However, analysis by random-effects model indicated a DL random-effect pooled OR = 0.67 [(95 percent CI 0.31 to 1.47); p = 0.32] with no significant association between the use of gabexate and the reduction of PEP (Figure 2).

Severe PEP was reported in two trials [8,9]. These two trials included 1172 patients with 6 patients suffering from severe PEP (5 in gabexate treatment group and 1 in the control group). The Q test of heterogeneity between studies was not significant (Q = 1.24, 1 degree of freedom, p = 0.27). The meta-analysis did not indicate association between gabexate use and reduction of severe PEP [IV fixed-effect pooled OR 3.78 (95 percent CI 0.62 to 22.98); p = 0.15] (Figure 2).

In addition, case-fatality ratio of PEP in these trials was extracted with report of case-fatality ratio in two trials [4,7]. The two trials included 1194 patients with 10 deaths in gabexate group and 6 in the control group. The Q test of heterogeneity of effect sizes was not significant (Q = 0.09, 1 degree of freedom, p = 0.76). Moreover, there was no significant association between the use of gabexate and the reduction of case-fatality ratio of PEP [IV fixed-effect pooled OR 0.68 (95 percent CI 0.19 to 2.43); p = 0.56] (Figure 2).

### Secondary outcome

Both post-ERCP hyperamylasemia and abdominal pain were considered as secondary outcome in the report. For post-ERCP hyperamylasemia, data were derived from all four RCTs [4,7-9]. These trials included 1783 patients with 700 patients suffering from post-ERCP hyperamylasemia. Among these patients, 335 patients were treated with gabexate and 365 patients with placebo. The Q test of heterogeneity of effect sizes was not significant (Q = 1.40, 3 degrees of freedom, p = 0.71). Although the post-ERCP hyperamylasemia was noted in 37.9% of patients with gabexate and in 40.6% of control patients, the results of the meta-analysis indicated no significant association between the use of gabexate and reduction of post-ERCP hyperamylasemia [IV fixed-effect pooled OR 0.88 (95 percent CI 0.72 to 1.07); p = 0.20] (Figure 2).

For post-ERCP abdominal pain, data were also extracted from four RCTs [4,7-9]. These trials included 1783 patients with 183 patients suffering from post-ERCP abdominal pain. Among these patients, 78 patients were in the gabexate group and 105 patients were in the control group. The Q test of heterogeneity of effect sizes was significant (Q = 9.21, 3 degrees of freedom, p = 0.03). Although the post-ERCP abdominal pain was noted in 8.8% of patients in gabexate group versus 11.7% of patients in control group, the results of meta-analysis showed that gabexate treatment in patients of post ERCP did not release abdominal pain as compared with placebo control [DL random-effect pooled OR 0.69 (95 percent CI 0.39 to 1.21); p = 0.19] (Figure 2).

### Sensitivity-analysis

**Figure 3:**
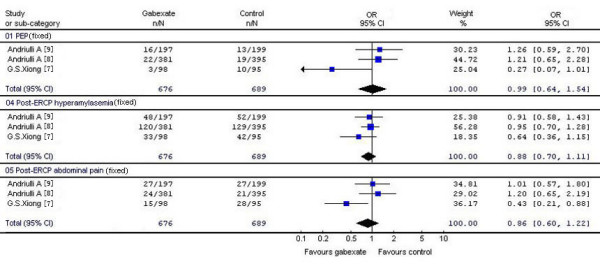
Sensitivity-analysis I: The study of the longest treatment duration [4] was excluded.

**Figure 4:**
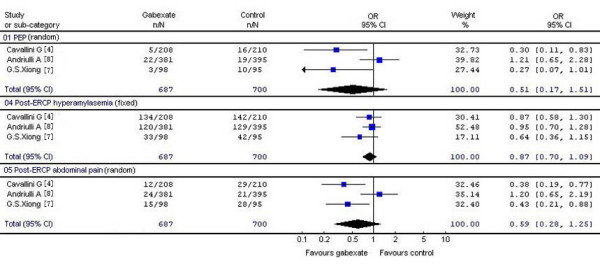
Sensitivity-analysis II: The study of the shortest treatment duration [9] was excluded.

In addition, we performed the sensitivity-analysis of these trials because treatment duration was one of the important factors that could influence the effectiveness of gabexate. With the sensitivity analysis, we excluded the longest [4] or the shortest treatment duration [9] separately. We did not include severe PEP and case-fatality ratio of PEP because of their limited sample sizes. As shown in Figure 3 and Figure 4, the overall estimates were virtually identical and the confidence intervals were similar between the sensitivity-analysis and the meta-analysis (Table 3).

### Publication bias

**Figure 5:**
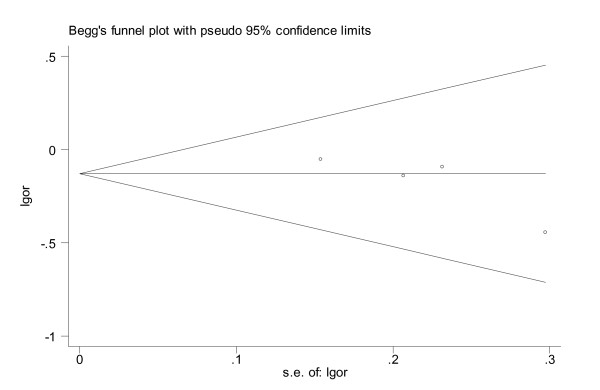
A funnel plot to explore publication bias.

Publication bias was assessed for all pooled ORs with confidence intervals using Begg's test [14,15]. It was shown as a funnel plot in Figure 5. No evidence of publication bias was found.

### Adverse effect

Adverse effects of gabexate were evaluated in current study. We found that two reports indicated different adverse effects of gabexate. In one report, there are 8 patients with adverse effects. Two patients were in gabexate group and six patients in placebo group [4]. Patients in gabexate group had mild nausea and vomiting in one case, and self-limiting dyspnea and a hypertensive crisis in another case. The six patients in the placebo group had different adverse events: nausea in two, vomiting in three, hypotension in one, sweating in one, and fatigue in one case. All these symptoms had been resolved without treatment. In another report from China, common symptoms such as bloating, nausea, vomiting, or fever were reported in both groups with no significant difference between gabexate treatment and placebo groups [7]. Therefore the authors concluded that there was no significant correlation between the use of gabexate and adverse effects.

## Discussion

Acute pancreatitis is the most frequent and serious complication of ERCP, which cannot be always avoided. At present, searching for drug's prevention of pancreatic injury after ERCP remains an important issue. Gabexate (a synthetic protease inhibitor) is able to inhibit the activities of several proteinases or peptidases such as kallikrein, trypsin, plasmin, thrombin, phospholipase A_2_, and C_1 _esterase. Since the activation of proteinase is one of the most important pathogeneses in pancreatic injury after ERCP, several studies have been reported the use of gabexate in post-ERCP for the prevention of pancreatitis. The first large-scale prospective study was conducted in Italy and its results were published in 1996 [4]. In this report, the authors had found that gabexate was able to reduce pancreatitis after ERCP as compared to the placebo (the occurrences of PEP were 2% versus 8% respectively). Moreover, a meta-analysis published in 2000 including six studies also reported that patients who received gabexate after ERCP had PEP at 1.6% of occurrence rate while patients in placebo group had PEP at 6.5% [16]. Therefore, they concluded that gabexate use was associated with a significant reduction of PEP (p < 0.001). Favourable conclusions concerning the use of gabexate for the prevention of post-ERCP hyperamylasemia and post-ERCP abdominal pain were also drawn in this meta-analysis.

Current study had collected four RCTs [4,7-9], which were published in the world with different languages and we evaluated the effectiveness and safety of gabexate in the prophylaxis of post-ERCP pancreatitis. The meta-analysis showed that the occurrences of PEP [OR = 0.67, 95%CI (0.31~1.47), p = 0.32], severe PEP [OR = 3.78, 95%CI (0.62~22.98), p = 0.15], the case-fatality ratio of PEP [OR = 0.68, 95%CI (0.19~2.43), p = 0.56], post-ERCP hyperamylasemia [OR = 0.88, 95%CI (0.72~1.07), p = 0.20], and post-ERCP abdominal pain [OR = 0.69, 95%CI (0.39~1.21), p = 0.19] did not correlate with the prophylactic use of gabexate. The results of meta-analysis indicated that gabexate could not prevent pancreatic injury after ERCP. Moreover, there was no association between the prophylactic use of gabexate and adverse effects although it was reported in two RCTs [4,7]. Further evaluation of the safety of gabexate in the prophylaxis of post-ERCP pancreatitis is required in the future. We had also evaluated the quality of these RCTs according to the Jadad score [10], and found that the results of meta-analysis were consistent with the sensitivity-analysis. However, the different conclusions between current study and previous publication [16] could be due to the selection criteria of inclusion and exclusion. In current study, we only included randomized controlled clinical trials while the other included clinical controlled trials and heavily relied on the conclusion of one clinical controlled trial [4] because other clinical controlled trials [5,6,17,18] had very small sample sizes. In addition, a recent meeting abstract of randomized controlled clinical trial (2006 Italian Digestive Week) also concluded that gabexate did not have beneficial effect on the prevention of pancreatic injury after ERCP [19]. Furthermore, discordance among the large randomized controlled trials was recognized and ascribed to heterogeneity of patients under study and differences in the experimental design [20]. The heterogeneity of patients and differences in the experimental design could explain the divergence of the results such as the inclusion of high risk patients with PEP [9] versus patients scheduled to undergo ERCP [4,7,8]. The duration of gabexate treatment could also contribute the different outcome, however, in current meta-analysis we did not include the duration of gabexate treatment in the evaluation. However, it was reported in a recent abstract that up to 12 hours infusion, gabexate still had no preventive effect on pancreatic injury after ERCP (2006 Italian Digestive Week).

## Conclusion

The present study shows no statistically significant benefit of prophylactic gabexate use for the prevention of PEP. Therefore it is not recommended that gabexate should be used in the prophylaxis of PEP routinely. Moreover it is clearly indicated that the adverse effect of gabexate after ERCP is required to be attention.

## Abbreviations

PEP: Post-endoscopic retrograde cholangiopancreatography pancreatitis. ERCP: Endoscopic retrograde cholangiopancreatography. OR: Odds ratio. CI: Confidence intervals. CBMdisc: China Biological Medicine Database.

## Competing interests

The author(s) declare that they have no competing interests.

## Authors' contributions

MZ: planning, data collection, study design and analysis, drafting and revising the manuscript. YC: conceiving of the study, participated in its design and helping to draft the manuscript. XY: data collection, study design and statistic analysis. JL: study design and analysis, and help to draft the manuscript. YZ: study design and analysis, and help to draft the manuscript. QZ: data collection, study design and statistic analysis. All authors read and approved the final manuscript.

## Pre-publication history

The pre-publication history for this paper can be accessed here:



## Figures and Tables

**Table 1:** Randomized controlled trials on the use of gabexate for the prevention of pancreatic injury after ERCP

Reference	Setting	Sample size	Patient inclusion criteria	Interventions	Outcomes	Allocation concealment
[4]	Italy multicentre	418	Patients over 18 years of age who were scheduled to undergo ERCP and, when indicated, endoscopic sphincterotomy	Gabexate 1 g given by intravenous infusion starting 30 to 90 minutes before endoscopy and continuing for 12 hours afterward	The incidence of PEP, post-ERCP hyperamylasemia, post-ERCP abdominal pain, case-fatality ratio of PEP	Adequate
[9]	Italy multicentre	396	Patients over 18 years of age who were scheduled to undergo ERCP and, when indicated, endoscopic sphincterotomy with one of the following "high risk" factors: 1) a non-dilated (< 8 mm in diameter) bile duct on pre-ERCP US 2) a history of gallstone-induced or post-ERCP pancreatitis 3) suspected sphincter of Oddi dysfunction	Gabexate 500 mg given by intravenous infusion starting 30 minutes before endoscopy and continuing for 2 hours afterward	The incidence of PEP, severe PEP, post-ERCP hyperamylasemia, post-ERCP abdominal pain	Adequate
[8]	Italy multicentre	776	Patients over 18 years of age who were scheduled to undergo ERCP and, when indicated, endoscopic sphincterotomy	Gabexate 500 mg given by intravenous infusion starting 30 before endoscopy and continuing for 6 hours afterward	The incidence of PEP, severe PEP, post-ERCP hyperamylasemia, post-ERCP abdominal pain, case-fatality ratio of PEP	Adequate
[7]	China single centre	193	Patients over 18 years of age who were scheduled to undergo ERCP and, when indicated, endoscopic sphincterotomy	Gabexate 300 mg given by intravenous infusion starting 30 before endoscopy and continuing for 4.5 hours afterward	The incidence of PEP, post-ERCP hyperamylasemia, post-ERCP abdominal pain	Adequate

**Table 2:** Jadad quality score of randomized controlled trials included in the meta-analysis

Reference	Randomization	Blinding	Withdrawals and dropouts	Jadad score
[4]	Centralized randomization schedule	Double blinding	Clearly reported	5
[9]	Computer-generated list	Double blinding	Clearly reported	5
[8]	Computer-generated list	Double blinding	Clearly reported	5
[7]	Computer-generated randomized set of numbers	Double blinding	Clearly reported	5

**Table 3:** Results of the meta-analysis and sensitivity-analysis

Outcome	Meta-analysis		Sensitivity-analysis I		Sensitivity-analysis II	
	OR	P	OR	P	OR	P
PEP	0.67 (0.31, 1.47)	0.32	0.99 (0.64, 1.54)	0.97	0.51 (0.17, 1.51)	0.22
Post-ERCP hyperamylasemia	0.88 (0.72, 1.07)	0.20	0.88 (0.70, 1.11)	0.29	0.87 (0.70, 1.09)	0.22
Post-ERCP abdominal pain	0.69 (0.39, 1.21)	0.19	0.86 (0.60, 1.22)	0.39	0.59 (0.28, 1.25)	0.17
